# Gene-Environment Interactions in Schizophrenia: A Literature Review

**DOI:** 10.3390/genes12121850

**Published:** 2021-11-23

**Authors:** Marah H. Wahbeh, Dimitrios Avramopoulos

**Affiliations:** Department of Genetic Medicine, Johns Hopkins University School of Medicine, Baltimore, MD 21205, USA; mwahbeh1@jhmi.edu

**Keywords:** schizophrenia, genetics, environment, gene-environment interactions, *Toxoplasma gondii*, cannabis, psychosis, polygenic risk score

## Abstract

Schizophrenia is a devastating mental illness with a strong genetic component that is the subject of extensive research. Despite the high heritability, it is well recognized that non-genetic factors such as certain infections, cannabis use, psychosocial stress, childhood adversity, urban environment, and immigrant status also play a role. Whenever genetic and non-genetic factors co-exist, interaction between the two is likely. This means that certain exposures would only be of consequence given a specific genetic makeup. Here, we provide a brief review of studies reporting evidence of such interactions, exploring genes and variants that moderate the effect of the environment to increase risk of developing psychosis. Discovering these interactions is crucial to our understanding of the pathogenesis of complex disorders. It can help in identifying individuals at high risk, in developing individualized treatments and prevention plans, and can influence clinical management.

## 1. Introduction

Schizophrenia is a chronic psychiatric disorder that affects ~20 million people worldwide [1,2]. The first episode of psychosis typically occurs in early adulthood, and the course of disease varies among individuals (Diagnostic and Statistical Manual of Mental Disorders, Fifth Edition, DSM-V). Patients with schizophrenia suffer an array of symptoms that are classified as positive, negative, and cognitive. Positive symptoms include delusions, hallucinations, and disorganized speech. Negative symptoms include flat affect and poverty of speech. Cognitive symptoms include impairments in attention, working memory, and executive functions (DSM-V). Many of these symptoms affect the functional independence of patients and often lead to social and/or occupational dysfunction. As a result, ~25% of schizophrenia patients suffer from clinical depression [3], substance abuse, and have a high risk of suicide [3,4], all of which make schizophrenia a major public health burden.

It has been well established that the interplay between an individual’s genetic makeup and the environment is important in the development of schizophrenia. Genetically, schizophrenia is highly heritable with heritability estimated at ~80%, and monozygotic twin concordance at ~45% [5]. The underlying genetic architecture of the disease is polygenic, involving both rare damaging variants (inherited and de novo) that highly increase risk and common variants with small to moderate effects [5]. In addition to genetics, exposure to different environmental factors at several stages during development (prenatal life, perinatal life, adolescence, and adulthood) have also been shown to contribute to the risk of developing schizophrenia [5]. Some of these environmental factors have cumulative/additive effects [6], and may be correlated with each other and possibly share causal pathways [7]. It is likely that the development of schizophrenia is the result of interactions between the two types of risk factors, genetic and environmental, rather than a result of their independent effects. This possibility has led to a considerable number of studies aiming to identify environmental risk factors and their likely interactions with the individual’s genetic background.

Uncovering and understanding the interactions between genetic and environmental risk factors during development is of great importance, as it will allow for the identification of disease related pathways that can be exploited to identify individuals at higher risk in the presence of certain exposures. This can influence clinical management and assist in creating individualized prevention plans for those at higher risk and treatment plans for patients. In this review, after a brief introduction on the genetic and environmental factors associated with schizophrenia, we showcase and discuss some of the most recent studies from the considerable volume of literature on the subject, connecting genetic risk to specific environmental exposures.

## 2. Genetic Risk of Schizophrenia

Although no single gene or variant has been identified that is sufficient to cause schizophrenia, some rare copy number variants (CNVs) and single nucleotide variants (SNVs) with high effect sizes have been implicated in the disease pathogenesis. Deletions at 22q11.2 represent some of the earliest discoveries [8] and mutations in the *SETD1A* gene are among the most recently identified variants [9]. Walsh et al. have shown that individuals with schizophrenia have more rare structural variants such as deletions and/or duplications of one or more genes compared to controls [10]. The use of large exome and genome sequencing datasets has allowed for identification of more rare coding variants that confer substantial risk for schizophrenia [11,12].

Common variants with smaller effect sizes have gained a lot of attention in the last decade as many have been identified after recent advancements in genome-wide association studies (GWAS), increasing our understanding of their contribution to the risk of disease [5,13]. The most recent schizophrenia GWAS to date identified 270 loci significantly associated with schizophrenia [13]. These loci include genes involved in dopamine signaling, glutamate signaling, ion channel function, and immune response [13,14]. Although these associations are common and of small or moderate effect, the large number of robust associations between DNA variants and schizophrenia is beginning to suggest possible ways to prevent and/or better treat the disease. These associations can also be used to estimate one’s risk through the calculation of a polygenic risk score (PRS), which sums the number of risk alleles weighted by their effect size. PRSs are valuable not only as a preliminary measure of risk, but also as a tool to assess genetic relationships of phenotypes and environmental exposures

Although schizophrenia is a complex disorder with environmental triggers playing a significant role in disease development, most genetic studies ignore the effect of the environment concentrating on genetic variation in isolation. Classic twin studies estimate the heritability of schizophrenia to be ~80% [5]. However, twin and adoption studies cannot fully account for shared environments (e.g., in utero), potentially overestimating the genetic contribution to disease development. It has also been shown that heritability estimates of schizophrenia are lower, at 64–67% [15,16,17] in family data from national records. Despite these potential overestimations of genetic risk, schizophrenia is clearly one of the most heritable neuropsychiatric disorders. Although that is the case, much of the heritability is still not explained by the additive effects of the known disease-associated genetic variants. Without assessing the environmental risk, we may miss pathophysiological pathways that are involved in disease development and thus hinder discovery of treatments and ways to prevent disease.

## 3. Environmental Risk Factors of Schizophrenia

Multiple biological, psychological, and social environmental risk factors have been associated with schizophrenia. They include infectious agents such as *Toxoplasma gondii* [18,19,20,21], cytomegalovirus [18,22,23,24], and others [18,23,24], inflammation markers such as cytokines and C-reactive protein [19], and obstetric complications including complications during pregnancy and delivery like bleeding, diabetes, rhesus incompatibility, preeclampsia, uterine atony, asphyxia, and emergency Cesarean section [25,26]. Additionally, increased risk has been associated with maternal nutritional deprivation [27,28] and cannabis use, especially during adolescence [29,30,31]. Psychosocial stress and childhood adversity have also been a major area of study linking the development of schizophrenia with an environmental exposure [32,33], along with a few studies that have associated increased risk with migration and urbanization (both of which could be considered causes of stress). Interestingly, season of birth also appears to play a role, with winter births being associated with higher risk [34,35,36,37]. It appears that the environmental impact on increasing schizophrenia risk is most prominent early in development (fetal period and childhood), although not exclusively, as some environmental exposures such as cannabis use have been shown to have an impact in adolescence and early adulthood. Environmental risk factors can function on an individual level or on a population and their effects can either be the direct culprit of the risk increase, or they may act as markers for other directly implicated factors not yet identified [37]. In comparison with genetic risk factors, individual environmental factors generally show higher odds ratios than common genetic variant detected by GWAS [38].

There are many limitations to studying environmental risk factors for schizophrenia and other neuropsychiatric disorders in general. Many environmental factors are very difficult to measure. Some are subjective experiences, like stress and childhood adversities. Others, like certain infections, have a limited impact window as they only have an effect during specific developmental stages. Some have variable dose-dependent outcomes, like cannabis use [39,40]. Many of the environmental factors may also be interdependent, which makes measuring their independent impact on psychosis and other psychiatric symptoms difficult [40]. The mechanisms through which environmental factors play a role in psychiatric disorders can also vary, as some operate within biological pathways through gene-environment interactions while others lead to changes in the epigenome impacting gene expression and consequently behavior [41]. Finally, different studies of the same outcome or exposure, use different models and variables adding yet another level of complexity to the interpretation of the data [40].

## 4. Gene-Environment (GxE) Interactions

While environmental factors are important and have relatively strong effects, the susceptibility of an individual to them will depend to a significant extent on the person’s genetic makeup. GxE interactions, although expected to exist widely, are difficult to identify because of the large search space, involving millions of variants and dozens of environments thereby diminishing statistical power. Nevertheless, several such interactions have been identified mostly through-hypothesis driven studies. In this review, we will specifically discuss studies addressing GxE interactions of genetic variation with infection, cannabis use, and psychosocial stress/childhood adversity as they are the most studied.

### 4.1. Infection

Epidemiological studies have long provided evidence for a link between specific infections and psychoses [19,42]. Prenatal maternal infections of *Toxoplasma gondii* (*Toxo*) [18,19], herpes simplex viruses [19,20,21], cytomegalovirus [18,22,23,24], and rubella [19] have been shown to increase risk of developing schizophrenia. These infectious agents seem to increase the risk by disrupting fetal neurodevelopment in response to infection as well as maternal immune activation [43,44,45]. Among them, *Toxo* is the most studied infectious agent with the strongest evidence to be implicated in schizophrenia development. It is a protozoan parasite that is neuro-invasive and causes toxoplasmosis. It can remain in the body of immune-competent mammalian hosts after infection for long periods of time. Prenatal maternal *Toxo* infections increase the risk of schizophrenia by 80% [46,47]. Multiple studies have assessed a potential GxE interaction between *Toxo* and schizophrenia-associated variants. In a meta-analysis of a toxoplasmosis susceptibility GWAS done by Wang et al., there was enrichment for genes associated with schizophrenia in populations with IgG seropositivity for *Toxo,* an indication of previous infection [48]. Although this shows that genes involved in schizophrenia development may also be involved in the susceptibility or the immune response to *Toxo*, conflicting evidence from a study by Lori et al. showed that PRS scores (based on schizophrenia GWAS data) do not predict schizophrenia in individuals who are IgG seropositive for *Toxo* [49]. The immune response and neuroinflammation due to *Toxo* and other infectious agents have also been implicated as a potential mechanism for GxE interactions. Avramopoulos et al. and Dickerson et al. showed that C-reactive protein (CRP), a peripheral marker of inflammation, was elevated in schizophrenia patients [50,51]. In a small case-control study, Mouhawess et al. investigated an association between polymorphism in Metallopeptidase-9 (MMP-9), *Toxo* infection and schizophrenia. They chose MMP-9 because of its role in neuroinflammation. They showed that the MMP-91562 C allele was only observed in patients diagnosed with schizophrenia and seropositive for *Toxo* IgG and IgM or IgG alone [52]. In another study, Ansari-Lari et al. tested 78 cases and 91 controls for a polymorphism in Glutathione S-Transferase Theta 1 (*GSTT1)* and its association with schizophrenia and *Toxo* infection [53]. *GSTT1* is located on 22q11.2, a locus previously associated with schizophrenia [8]. They concluded that risk of schizophrenia increased in patients who were infected with *Toxo* and had a deletion in *GSTT1*, although they also mention that other studies have not shown an association between variants in *GSTT1* and schizophrenia [53]. Severance et al. investigated the association of complement C4 gene copy number and haplotype groups with schizophrenia and *Toxo* IgG along with other biomarkers of pathogen exposure. The C4 gene is structurally complex with different haplogroups that reflect copy number variations. C4 gene forms include C4A gene short (*C4AS*), C4A gene long (*C4AL*), C4B gene short (*C4BS*), and C4B gene long (*C4BL*). The authors found that the haplogroup containing two copies of 4AL had a strong association with schizophrenia and *Toxo* IgG [54].

There have been a few studies investigating genetic interactions with herpes simplex viruses, HSV-1 and -2. It is known that human leukocyte antigen (HLA) gene polymorphisms, located on chromosome 6, impact immune surveillance. With that in mind, Bamne and colleagues tested schizophrenia-associated SNPs on chromosome 6p for association with exposure to HSV-1. They found that HSV-1 exposure was significantly associated with one of the tested schizophrenia-associated SNPs (rs3130297), yet the HSV-1 associated allele was different from the schizophrenia-associated allele. They hypothesize that this could be a result of epistatic effects at the SNP rs3130297 or another SNP in linkage disequilibrium (LD) [55]. Demontis et al. investigated an association between schizophrenia associated SNPs in *GRIN1*, *GRIN2A*, *GRIN2B*, *GRIN2C*, and *GRIN2D,* exposure to HSV-2 during pregnancy, and schizophrenia development in offspring in a case control study with 984 patients and 1500 controls. *GRIN1*, *GRIN2A*, *GRIN2B*, *GRIN2C*, and *GRIN2D* all encode subunits of the N-methyl-D-aspartate receptor (NMDAR), which has been shown to be involved in the development of schizophrenia. They genotyped a total of 81 tag SNPs (tSNPs), which are defined as SNPs not in LD representing different haplotypes and investigated their association with maternal HSV-2 seropositivity. They showed a significant interaction between maternal HSV-2 seropositivity and SNPs rs1805539 and rs1806205 in the gene *GRIN2B* in the offspring [56]. Moreover, Pandy et al. evaluated immunoglobulin GM (γ marker) allotype associations with schizophrenia. GM alleles have been shown to impact immunity to HSV-1 and other viruses. In their analysis, they found that individuals with the GM genotypes 3/3; 23−/23− were more than three times more likely to develop schizophrenia than individuals with other genotypes. These results implicate immunoglobulin GM genes in schizophrenia and suggest a potential GxE interaction pathway for viruses like HSV-1 [57]. Shirts et al. studied five selected SNPs from a set of tSNPs in the 100 kb region flanking the marker D6S2672 in the gene *MICB*, previously associated with schizophrenia. They performed their interaction analysis between genes, seropositivity of HSV-1 and schizophrenia on two separate samples (one was a case-control cohort, and one was a case-parent trio cohort). They found two SNPs associated with both seropositivity for HSV-1 and schizophrenia in non-schizophrenia samples and suggested as a possible interpretation of this result an interaction between genotype and HSV-1 seropositivity [58]. In another study, the same group showed that SNPs rs2272127 and rs11465702 at the gene *IL18RAP* were associated with HSV-1 seropositivity in patients with schizophrenia [59]. All of the mentioned studies are candidate gene/variant studies, and experience has shown that publication bias and low power in such studies can sometimes lead to false positives. More research is still needed to further investigate the link between herpes simplex viruses, schizophrenia and the genome as no genome wide studies employing PRS scores have been done to our knowledge.

Most recently, the COVID-19 pandemic impacted over 20 million people who became infected with SARS-CoV-2 [60]. It has been shown that some COVID-19 patients experience neuropsychiatric symptoms after infection [61,62]. Moni et al. investigated the potential interactions between SARS-CoV-2 and underlying genetic psychiatric susceptibilities [60]. They analyzed the transcriptome of peripheral blood mononuclear cells from COVID-19 patients and found elevated expression of inflammatory cytokine and interferon response genes [60]. It has also been shown that SARS-CoV-2 can infect nerve cells [60]. Cytokine storm is a common consequence of SARS-CoV-2 infection and a cause of complication and poor prognosis for many COVID-19 patients [63]. Patients also have elevated levels of cytokines and chemokines like interleukin-6 (IL-6), interleukin-8 (IL-8), and interleukin-10 (IL-10) after infection [62,64]. This is of interest, as it has been shown that inflammation poses an environmental risk to increasing risk of schizophrenia [19]. Elevated IL-8 during the second trimester and the early third trimester in mothers increased risk for schizophrenia development in the offspring [19,65]. To our knowledge, no studies have directly associated infection with SARS-CoV-2 with an increased risk for psychosis, however, investigating why some COVID-19 patients suffer neuropsychiatric symptoms after infection may be an interesting area of research.

### 4.2. Cannabis Use

Evidence over the last few decades has suggested that cannabis use in early adolescence is involved with the development of psychosis [29,30,31,66]. A mendelian randomization study by Vaucher et al. suggested that the association between cannabis and increased risk of schizophrenia is causal [31]. While one must be cautious because mendelian randomization is sensitive to pleiotropy, where the variants associated with cannabis use may be associated with some other variable that is the true cause of risk, the authors tested and found no such evidence, contingent on the power of the test. Given the relatively large proportion of the population that has ever used or currently uses cannabis, use alone is not sufficient to cause schizophrenia. Several studies, from candidate gene studies to genome wide studies, have all provided evidence for interplay between genes and cannabis use in the development of psychosis and schizophrenia [67]. McGuire et al. assessed for family history of schizophrenia in 23 cannabis users and 46 controls, where ⅔ of the individuals in each group had a psychosis diagnosis. They found that individuals who developed psychosis after cannabis use were more likely to have family history of schizophrenia, showing that experiencing psychotic symptoms after cannabis use may be due to a potential genetic predisposition [30,68].

One of the most studied genes for its interaction with cannabis and impact on schizophrenia development is *COMT*. *COMT* codes for catechol-O-methyltransferase, an enzyme that plays a role in dopamine breakdown. It is also localized to chromosome 22q11.1. A longitudinal study following individuals to adulthood looked into a specific polymorphism (Val158Met) in *COMT*, as a potential gene moderator for the association between cannabis use and psychosis development [69]. The results of the study implicated the *COMT Val158Met* Val allele as a link between adolescent cannabis use and an increased risk for later psychosis [69]. Although other studies have found conflicting evidence [70], Henquet et al. reported similar findings to the Caspi et al. study [71]. They followed adult-onset cannabis users with psychosis and controls, and showed that individuals homozygous for the Val allele at *COMT Val158Met* were more sensitive to the effects of delta-9-tetrahydrocannabinol (THC), the main psychotropic component in cannabis, on cognition and psychosis [71].

Aside from *COMT*, cannabis users with specific alleles in variants in the *DRD2* [72], *FAAH* [73], and *AKT1* genes [74,75] have been found to be much more likely to develop psychotic symptoms. It is important to note, however, that in 2020, Hindocha et al. reported contradictory results with a larger sample, showing that neither *COMT*, *AKT1*, or *FAAH* modulated a psychosis response to cannabis use [76]. Cannabinoid receptor 1 (CB1) has also been implicated in cannabis use and schizophrenia risk [77,78] although, again, with conflicting evidence. CB1 is a G-coupled protein receptor involved in neurotransmission of glutamate, dopamine, and γ-aminobutyric acid (GABA), and is expressed in the brain [79]. CB1 is stimulated by endocannabinoids and exogenously by THC. CB1 has been shown to both be activated and blocked by THC [77,80,81]. Ujiki et al. found that an AAT repeat polymorphism in the 3′ region of *CRN1*, the gene encoding CB1, was significantly associated with hebephrenic schizophrenia in the Japanese population [82]. Ho et al. genotyped 12 tSNPs [83], and although none showed significant associations with either patients who were cannabis users or nonusers, 3 of the tSNPs had significant associations with total cerebral white matter volumes in patients. Moreover, they showed that patients with heavy cannabis use had smaller frontotemporal white matter volumes compared to patients not using cannabis [83]. Other studies investigating SNPs in *CRN1* have shown no associations between the variants and schizophrenia in relation to cannabis use. Examples include a study by Seifert et al. where they performed an association study of three different *CNR1* polymorphisms and did not detect statistically significant associations in either the case or control groups [84]. Another study looked for associations with *CNR1*, including four SNPs (one of which was in common with the Seifert et al. study), and also saw no significant association between schizophrenia and any of the tested SNPs, even when they included cannabis use as a dependent variable in their regression analysis [77].

In the last few years, studies employing genome wide data and PRS scores have emerged, adding to the evidence of GxE interactions linking cannabis use and schizophrenia. In a study of 1574 participants, French et al. found that in male cannabis users, high PRS scores for schizophrenia were associated with low cortical thickness, a known risk factor for psychosis. This indicates that cannabis use could moderate the association between the genetic risk and cortical maturation in schizophrenia patients [85]. In another study, Wainberg et al. assessed the relationship between self-reported psychotic experiences, cannabis use, and PRS scores for schizophrenia in participants from the UK BIOBANK [86]. They found that 7% of those who were cannabis users self-reported psychotic symptoms compared to only 4.1% of those who have never used cannabis. The effect of cannabis was dose-dependent, as 8.4% of monthly users, 8.8% of weekly users, and 9.6% of daily users reported psychotic experiences. They also found that cannabis users with the highest PRS scores for schizophrenia had 1.58-fold greater adjusted odds of psychotic experiences (self-reported) compared to 1.39-fold in users with the lowest PRS scores [86]. Another group also found that there was an additive interaction between schizophrenia PRS scores and regular cannabis use [87].

The controversy around whether cannabis use has a direct causal relationship to increasing schizophrenia risk remains. Patients with schizophrenia may experience higher levels of stress and depressive symptoms. Cannabis has been known to help alleviate stress and improve symptoms [88,89], making it plausible that schizophrenia patients are more likely to use cannabis as a form of self-medication. A longitudinal study by Hiemstra et al. found that individuals with high PRS scores for schizophrenia were more likely to use cannabis in adolescence (ages 16–20 and using stringent PRS thresholds) [90]. In another study by Verweij et al., PRS scores for schizophrenia were significantly associated with lifetime and regular cannabis use, with risk scores explaining up to 0.5% of the variance [91]. Power et al. also used PRS scores for schizophrenia to test if they correlate with cannabis use and found positive associations [92]. These studies add complexity to the association between cannabis use and schizophrenia, and show that the link may not be as straight forward.

### 4.3. Psychosocial Stress and Childhood Adversity

Psychosocial stress and childhood adversity have been shown to increase risk for psychosis and schizophrenia. Psychosocial stress can be defined as stress that is caused by perception of social threat that results in emotional tension and discomfort. This includes situations of perceived social evaluation and social exclusion as well as stressful life events [32,93]. Childhood adversity is defined by exposure to abuse, neglect, or family dysfunction [33]. The earliest studies investigating a link between genes, stress, and psychosis development were candidate gene studies. Winkel et al. investigated GxE interactions between stress and *COMT* (already implicated in cannabis use and schizophrenia) after epidemiological studies had shown that individuals who were homozygous for the Met allele at the *COMT Val158Met* were more sensitive to stress. In a small sample, Winkel et al. showed that patients with a psychotic disorder who were carriers of the Met allele had more psychotic experiences in response to daily stress compared to controls, implicating *COMT* in stress and psychosis [94]. Collip et al. followed up on the Winkel et al. study with a larger sample and confirmed a significant interaction between *COMT* Met allele carriers, stress, and psychosis [95]. Interestingly, a study by Stefanis et al. investigated the interaction between *COMT Val158Met* and psychosis in a sample of young men entering compulsory military training, and found that exposure to stress at army induction was associated with psychotic symptoms and that carriers of the *COMT* Val allele were more susceptible to the effect of stress, contradicting data from the previous studies [96,97]. *COMT* has also been investigated as a moderator between childhood adversity and psychosis [98]. Debost et al. found that *COMT* Val allele carriers who were exposed to childhood adversity and were also carriers of *MTHFR* T alleles were at increased risk of schizophrenia compared to controls [98]. Brain-Derived Neurotrophic Factor (*BDNF*) [96], *CACNA1C* [99], *NRG1* [100], and *FKBP5* [101] are all additional candidate gene examples of a genetic link between stress, childhood adversity, and psychosis.

Genome wide studies employing PRS scores have also investigated genes, stress, and childhood adversity interactions and effects on psychosis. A 2009 study by Tessner et al. followed adolescent individuals with schizotypal personality disorder (SPD) and used that as a proxy for elevated genetic risk for psychotic disorders since SPD symptoms are similar to prodromal signs of schizophrenia. They found that among adolescents, including those who they deemed at high risk for developing a psychotic disorder, the frequency of daily stressors in a 24-h period predicted an increase in positive prodromal symptoms one year later. Although the daily stressors did not correlate with SPD in predicting prodromal symptoms in relation to stress, they did report that adolescents with SPD had stronger perceptions of stress to the same daily stressors than their non-diagnosed peers [102]. Pries et al. used schizophrenia PRS scores of a twin cohort and their siblings of a final sample of 593 participants to investigate a GxE interaction between the scores, childhood adversity or daily stressors and psychosis. Their study concluded that there is an interaction between PRS scores and childhood adversity affecting subtle psychosis expression and stress-sensitivity. They hypothesize that sensitivity to daily stressors is affected by previous exposure to bigger stressors like childhood adversity, highlighting that the type, timing, and severity of stress has a potential role in psychosis development [103]. The same group investigated a sample of 1699 Schizophrenia patients and 1542 unrelated controls from the European Network of National Networks studying GxE Interactions in Schizophrenia (EUGEI), and found evidence of an additive interaction between schizophrenia PRS scores and childhood bullying, emotional abuse, sexual abuse, and emotional neglect, all of which are examples of childhood adversity [87]. In another study Hatzimanolis et al. studying a young healthy male population found that the observed association between polygenic risk for schizophrenia and schizotypal traits is modified by stress [104]. In a 2020 study of 6646 participants, Pries et al. calculated PRS scores as well as exposome scores for schizophrenia. They define the exposome score as a cumulative measure of environmental liability for schizophrenia [105]. In their analysis investigating the associations of stressful life events with either the exposome score or the PRS score, they found that the association of stressful life with mental outcomes was moderated only by the exposome score and not the PRS score [106].

It’s important to note that many environmental factors can be considered sources of psychosocial stress and likely are also associated with increased schizophrenia risk. Winkel et al. have summarized a few studies on indirect measures of stress like urbanicity [96], migrant status, and others.

## 5. Conclusions

The studies discussed in this review suggest that investigating the link between environmental and genetic risks of schizophrenia through GxE interaction studies can contribute to our understanding of schizophrenia development. Despite the importance of these studies and the need for more, especially more agnostic, genome-wide exploration studies of GxE interactions in schizophrenia, performing them has challenges and limitations [105]. Many of the earliest studies of GxE interaction in schizophrenia used family history as a proxy for genetic risk, and although it is strongly related, family history is not equivalent to genetic contributions [106]. The results of more recent studies employing PRS scores can’t be generalized to all ethnicities, as PRS for schizophrenia are only predictive for populations genetically close to those used to calculate the scores. GxE interaction studies also require large sample sizes compared to genetic or environmental studies, which can be challenging [46]. Moreover, questions of possible confounding by GxE correlation [72] and the difficulty in reliably measuring environmental factors complicates how we can interpret data from GxE interaction studies.

Although GxE interaction studies are difficult to conduct for reasons mentioned above, studying GxE interactions in schizophrenia and other psychiatric disorders remains essential, as such knowledge can enhance our approaches to diagnosis and treatment, for which there is a strong need for this devastating disorder.
